# Genomic resources and draft assemblies of the human and porcine varieties of scabies mites, *Sarcoptes scabiei* var. *hominis* and var. *suis*

**DOI:** 10.1186/s13742-016-0129-2

**Published:** 2016-06-02

**Authors:** Ehtesham Mofiz, Deborah C. Holt, Torsten Seemann, Bart J. Currie, Katja Fischer, Anthony T. Papenfuss

**Affiliations:** Bioinformatics Division, The Walter and Eliza Hall Institute of Medical Research, Parkville, VIC 3052 Australia; Department of Medical Biology, University of Melbourne, Melbourne, VIC 3010 Australia; Menzies School of Health Research, Charles Darwin University, Casuarina, NT 0811 Australia; Victorian Life Sciences Computation Initiative, University of Melbourne, Melbourne, VIC 3010 Australia; QIMR Berghofer Medical Research Institute, 300 Herston Road, Herston, QLD 4006 Australia; Sir Peter MacCallum Department of Oncology, University of Melbourne, Melbourne, VIC 3010 Australia; Peter MacCallum Cancer Centre, Melbourne, VIC 3000 Australia

**Keywords:** Scabies mite, *Sarcoptes scabiei* var. *hominis*, *Sarcoptes scabiei* var. *suis*, Indigenous Australian health

## Abstract

**Background:**

The scabies mite, *Sarcoptes scabiei,* is a parasitic arachnid and cause of the infectious skin disease scabies in humans and mange in other animal species. Scabies infections are a major health problem, particularly in remote Indigenous communities in Australia, where secondary group A streptococcal and *Staphylococcus aureus* infections of scabies sores are thought to drive the high rate of rheumatic heart disease and chronic kidney disease.

**Results:**

We sequenced the genome of two samples of *Sarcoptes scabiei* var. *hominis* obtained from unrelated patients with crusted scabies located in different parts of northern Australia using the Illumina HiSeq. We also sequenced samples of *Sarcoptes scabiei* var. *suis* from a pig model. Because of the small size of the scabies mite, these data are derived from pools of thousands of mites and are metagenomic, including host and microbiome DNA. We performed cleaning and *de novo* assembly and present *Sarcoptes scabiei* var. *hominis* and var. *suis* draft reference genomes. We have constructed a preliminary annotation of this reference comprising 13,226 putative coding sequences based on sequence similarity to known proteins.

**Conclusions:**

We have developed extensive genomic resources for the scabies mite, including reference genomes and a preliminary annotation.

**Electronic supplementary material:**

The online version of this article (doi:10.1186/s13742-016-0129-2) contains supplementary material, which is available to authorized users.

## Data description

The scabies mite, *Sarcoptes scabiei*, is an ectoparasitic acari, which causes rashes and extreme itching - known as scabies in humans. Different varieties of the scabies mite also cause mange in other species of mammals including domestic animals, livestock and wildlife. Scabies is known to cause significant morbidity in some populations, in particular Indigenous communities in Australia. We present extensive genomic sequencing data from human (*Sarcoptes scabiei* var. *hominis*) and pig (*Sarcoptes scabiei* var. *suis*) varieties of scabies mites, including Illumina whole genome sequencing data from two independent samples of adult scabies mites collected at different times from human patients from different regions of northern Australia, and from four samples of scabies mites from a pig model collected at different times and washed using different protocols to reduce bacterial contamination from host skin and mite gut. We created draft genome assemblies for var. *hominis* and var. *suis* from these resources.

### Samples and sequencing

Scabies mites (var. *hominis*) were individually picked from skin scrapings collected 14 months apart from two unrelated patients from two different regions of northern Australia with severe crusted scabies (Patients A and B). Over 1000 mites were collected in each sample. Two pig mange mite (var. *suis*) samples were collected from an inbred population of mites from a pig model [1]. The first sample consisted of >1000 mites from adult, nymph, larva and egg life stages (Pig Unwashed). The second sample, also containing all life stages, was split into three subsamples that were washed - to reduce the amount of bacteria present on the surface of the mites owing to the wound micro-environment - using three different protocols (Pig Washed 1, 2 and 3): (i) 15 min wash at room temperature in 4 % paraformaldehyde in water [2]; (ii) 1 h incubation at 37 °C in 150 mM NaCl, 10 mM EDTA pH8.0, 0.6 % SDS and 0.125 μg/μl lysozyme [3]; (iii) 1 h incubation at 37 °C in 1 % bleach (sodium hypochlorite) in water. In all protocols, mites were subsequently rinsed twice in water. Between wash steps, mites were centrifuged at 10,000 rpm for 2 min.

Whole mites were crushed and DNA was extracted from each sample using a QIAGEN Blood and Cell Culture DNA Kit and a modified procedure adapted from the manufacturer’s protocol. Washed mites were submerged in 1 ml of ice-cold lysis buffer (20 mM EDTA, 100 mM NaCl, 1 % TritonX-100, 500 mM guanidine-HCl, 10 mM Tris pH7.9) and homogenized with stainless steel beads of 2.8 mm diameter at 6800 rpm, three cycles, 30 s per cycle, and 30 s between cycles. The suspension of lysed mites was supplemented with DNase-free RNase A to 0.2 mg/ml and with proteinase K to 0.8 mg/ml and incubated at 50 °C for 1.5 h. After centrifugation at 4000 × *g* for 10 min to pellet insoluble debris, the genomic DNA was isolated on the QIAGEN genomic tip as per the manufacturer’s protocol. Six DNA libraries were constructed and 100-base pair (bp) paired-end reads were generated using an Illumina HiSeq 2500 (see Table 1 for details).

**Table 1:** Details of sequencing libraries

Sample type	Label	Washing protocol	Number of read pairs
Clinical isolate	Patient A	-	53,699,468
Clinical isolate	Patient B	-	45,851,518
Lab model	Pig Unwashed	-	59,011,146
Lab model	Pig Washed 1	Paraformaldehyde	62,090,067
Lab model	Pig Washed 2	Lysozyme	56,485,415
Lab model	Pig Washed 3	Bleach	55,580,620

### Genome assembly

Read qualities were assessed using FASTQC [4], and reads were adapter- and quality-trimmed (Q ≥ 20) using Trim Galore! (v3.0.1) [5].

Preliminary *de novo* assemblies of the adapter- and quality-trimmed reads of the Patient A, Patient B and Pig Unwashed samples were performed by using Velvet (v1.2.08) [6]. For the Patient B library, k-mer values of 61, 63, 65, 67, 69, 71, 73, 75, 79, 85, 89 and 95 were used. For the Patient A and three Pig Unwashed libraries, k-mer values of 69, 75, 77, 79, 81, 83, 85, 89 and 95 were used. The best assemblies (assessed using the scaffold N50) were obtained with a k-mer of k = 77 (Patient A, N50 = 27.4 kb), k = 63 (Patient B, N50 = 36.0 kb) and k = 81 (Pig Unwashed, N50 = 7.5 kb) (see Additional file 1 for details). Platanus (version 1.2.1) [7] was also used to perform a preliminary assembly of all six libraries, producing assemblies with better scaffold N50 values (*GigaScience* repository [8] for var. *suis*).

Since the scabies mite is a tiny, obligate parasite, it is difficult to avoid contamination from the host and from host skin and mite gut microbiomes. In addition, it was necessary to sequence thousands of intact mites, which incorporated the mite gut. Reads from the host genome were removed *in silico* from each sample using Bowtie 2 (version 2.2.5) [9]. Human hg19 and pig susScr3 reference genomes from the University of California, Santa Cruz, were used to build Bowtie 2 reference indices for alignment. For each sample, adapter- and quality-trimmed reads were aligned to the host reference genome using Bowtie 2 (using mode ‘--end-to-end’ and parameter ‘--very-fast’). The proportion of reads aligning to host reference genomes varied from 11 to 56 % (Table 2). Non-host reads were extracted from the alignment SAM files using the SAMtools [10] ‘view’ command with flag ‘-f 12’ (read unmapped, mate unmapped).

**Table 2:** Summary statistics for host-filtered Platanus assemblies

	Patient A	Patient B	Pig unwashed	Pig washed 1	Pig washed 2	Pig washed 3	Pig washed pooled
Host filtering using Bowtie 2 alignment							
Host-aligned read percentage	55.68 %	22.51 %	14.20 %	10.99 %	43.98 %	11.07 %	N/A
Scaffolds							
Scaffold N50	29,787	45,917	6352	6835	22,475	36,156	4883
Largest scaffold	509386	794311	88,812	681,477	423,133	809,115	299,570
Total assembled bases	68,937,519	61,661,613	69,459,333	68,875,212	61,832,214	56,344,534	75,837,484
No of scaffolds	99,178	66,591	47,952	149,238	83,245	26,086	212,580
Scaffolds (≥500 bp)							
Major scaffold N50	43,122	62,417	7574	17,034	30,929	40,825	-
Largest scaffold	509,386	794,311	88,812	681,477	423,133	809,115	-
Total assembled bases	56,795,385	53,697,990	62,853,857	47,516,449	52,301,800	53,472,496	-
No of scaffolds	4276	3157	17236	7586	5102	4269	-

Each host-filtered library was then assembled using Platanus (version 1.2.1, default settings), because this method performed better in the preliminary assembly of unfiltered reads. This produced assemblies with scaffold N50s ranging from 6 kb (Pig Unwashed) to 46 kb (Patient B) and major N50s up to 62 kb (see Table 2 for details). A pooled assembly of the three host-filtered washed pig samples (Pig Washed 1, 2 and 3) was also performed, producing an N50 of 4.8 kb.

The Platanus assemblies of Patient B and Pig Washed 3 had the largest major N50s (62.4 kb and 40.8 kb respectively) and were selected as the var. *hominis* and var. *suis* draft reference genomes (Table 3).

**Table 3:** Summary statistics for *Sarcoptes scabiei* draft reference genomes

Genome	Assembly size (bp)	No of scaffolds	Major scaffold N50 (bp)	Largest scaffold (bp)	No of gene features annotated
*Sarcoptes scabiei* var. *hominis* (Patient B)	53,667,537	3138	63,351	794,311	13,226
*Sarcoptes scabiei* var. *suis* (Pig Washed 3)	53,470,956	4268	40,825	809,115	-

These two draft assemblies were then filtered for bacterial scaffolds by aligning scaffolds to the National Center for Biotechnology Information (NCBI) Microbial RefSeq database v72 [11] using BLASTN (version 2.2.30+; E-value cutoff 10^−20^; max_target_seqs = 1) [12]. The best hits in which >80 % of the scaffold length aligned to bacterial sequences were filtered out, removing 19 scaffolds from Patient B and one scaffold from Pig Washed 3. A similar search on the assemblies prior to filtering small contigs showed that most of the bacterial contigs in the assemblies were shorter than 500 bp.

To estimate the proportion of bacterial DNA contaminating the samples, microbial classification was performed on unfiltered reads from each sample using Kraken [13]. Kraken was run with default settings using the standard bacterial, archaeal and viral database (downloaded on 3 November 2014). The samples were found to have contaminant proportions of between 4 and 9 % (Additional file 2).

After removal of bacterial scaffolds, we obtained the final *Sarcoptes scabiei* var. *hominis* and var. *suis* draft genome assemblies, which had final major scaffold N50 values of 63.3 kb (Patient B) and 40.8 kb (Pig Washed 3). The genome sizes of the assemblies were 53.7 Mb in 3138 scaffolds (Patient B) and 53.5 Mb in 4268 scaffolds (Pig Washed 3) (Table 3). Protocols presented here are also available in protocols.io [14].

### Estimation of genome completeness

To estimate the completeness of the assemblies, the Core Eukaryotic Genes Mapping Approach (CEGMA) [15] and Benchmarking Universal Single-Copy Orthologs (BUSCO) [16] strategies were applied to the var. *hominis* and *suis* draft genome assemblies. CEGMA (v2.5) was run with default settings on both assemblies to estimate genome completeness based on 248 ultra-conserved core eukaryotic genes (CEGs) found in nearly all eukaryotes. For both assemblies, CEGMA estimated 98.79 % completeness based on complete matches and 99.19 % completeness based on partial matches. BUSCO (v1.1b) was run in default settings using single-copy ortholog gene set databases for eukaryote taxonomic group. Seventy-five percent (75 %) of genes from the gene set of eukaryotes were predicted in both the draft genomes (66 % complete and 8.8 % fragmented genes in var. *hominis* and 67 % complete and 7.9 % fragmented in var. *suis*).

### Preliminary genome annotation

A preliminary annotation of the var. *hominis* draft genome (Patient B) assembly was constructed by aligning UniProtKB/Swiss-Prot proteins (release 2015_07) [17] with the assembly using TBLASTN (version 2.2.30+; E-value cutoff 10^−6^) [12]. Multiple annotations intersecting scaffold positions on the same strand were merged into a single annotation using the BEDTools (v2.25.0) [18] ‘merge’ sub-command in strand-specific mode. After the merging step, a total of 13,226 gene features were annotated.

### Comparison with other scabies genomics resources

The mitochondrial genome reference sequence for *Sarcoptes scabiei* var. *hominis* and var. *suis* have been published [19] and used to investigate within-patient diversity of infestations*.* A draft genome assembly of *Sarcoptes scabiei* var. *canis* is also available [20]. The scaffold N50 of this genome was 11.6 kb with a largest scaffold of 358.8 kb; the total assembly size was 56.2 Mb with a total of 18,600 scaffolds. In comparison, the var. *hominis* (Patient B) draft assembly had a scaffold N50 of 63.3 kb with a largest scaffold of 794.3 kb; the total assembly size was 53.6 Mb with a total of 3138 scaffolds. The annotation of the var. *canis* genome consisted of 10,644 predicted protein-coding genes, and the preliminary annotation of the var. *hominis* genome consists of 13,226 gene similarity features. The var. *canis* assembly had an estimated completeness of 93.55 % using CEGMA, while both var. *hominis* and var. *suis* draft genome assemblies had 99.19 and 98.79 % completeness based on partial and complete matches respectively.

## Availability of supporting data

Supporting data is available in the *GigaScience* repository [8] and raw data in NCBI (BioProject accession: PRJEB12428). Genome assembly protocols presented here are also archived in protocols.io [14].

## Ethics approval and consent to participate

The collection of human patient samples was approved by the Human Research Ethics Committee of the Northern Territory Department of Health and Menzies School of Health Research (approval 13–2027), and informed consent was obtained from each participant. Animal care and handling procedures used in this study followed the Animal Care and Protection Act, in compliance with the Australian code of practice for the care and use of animals for scientific purposes, outlined by the Australian National Health and Medical Research Council (NHMRC). The study was approved by the Queensland Animal Science Precinct (QASP) and the QIMR Berghofer MRI Animal Ethics Committees (DEEDIAEC SA2012/02/381, QIMR A0306-621 M).

## Additional files

Additional file 1: Summary statistics from preliminary assemblies using Velvet. (XLSX 10 kb) Additional file 2: Kraken classification of raw, unfiltered reads. (XLSX 8 kb)

## Abbreviations

bp: base pairs
BUSCO: Benchmarking Universal Single-Copy Orthologs
CEGMA: core eukaryotic genes mapping approach
CEGs: core eukaryotic genes
NCBI: National Center for Biotechnology Information
